# Comparison of intraoperative blood loss during spinal surgery using either remifentanil or fentanyl as an adjuvant to general anesthesia

**DOI:** 10.1186/1471-2253-13-46

**Published:** 2013-12-05

**Authors:** Hiroaki Kawano, Sawa Manabe, Tomomi Matsumoto, Eisuke Hamaguchi, Michiko Kinoshita, Fumihiko Tada, Shuzo Oshita

**Affiliations:** 1 Department of Anesthesiology and Clinical Research, National Hospital Organization Zentsuji Hospital, Zentsuji, Japan; 2 Current affiliation: Department of Anesthesiology, Tokushima Prefectural Central Hospital, Tokushima, Japan; 3 Department of Anesthesiology, Kagawa National Children’s Hospital, Zentsuji, Japan; 4 Department of Anesthesiology, Tokushima University Hospital, Tokushima, Japan

**Keywords:** Intraoperative blood loss, Remifentanil, Hemodynamics, Fentanyl, Spinal surgery, General anesthesia

## Abstract

**Background:**

Remifentanil enhances intraoperative hemodynamic stability, suggesting that it may decrease intraoperative blood loss when included as an adjuvant to general anesthesia. This retrospective study compared intraoperative blood loss during spinal surgery in patients administered either remifentanil or fentanyl as an opioid adjuvant.

**Methods:**

We reviewed clinical and surgical data from 64 consecutive laminoplasty or laminectomy patients treated at National Hospital Organization Zentsuji Hospital between April 2010 and March 2011. Patients received either remifentanil (n = 35) or fentanyl (n = 29) as an opioid analgesic during general anesthesia. In addition to intraoperative blood loss, indices of hemodynamic stability, including heart rate as well as systolic, mean, and diastolic blood pressure (BP), were compared over the entire perioperative period between remifentanil and fentanyl groups.

**Results:**

The remifentanil group exhibited significantly lower intraoperative arterial BP than the fentanyl group. Intraoperative blood loss was also significantly lower in the remifentanil group (125 ± 67 mL vs. 165 ± 82 mL, *P* = 0.035).

**Conclusions:**

Intraoperative blood loss during spinal surgery was decreased in patients who received remifentanil as an opioid adjuvant, possibly because of lower intraoperative BP. A larger-scale prospective randomized controlled trial is warranted to confirm our results and to test whether remifentanil can decrease intraoperative blood loss during other surgical procedures.

## Background

Remifentanil, an ultra-short-acting phenylpiperidine opioid analgesic agent, is widely used for general anesthesia because of its unique pharmacokinetic profile. Large doses of remifentanil can be administered to attenuate endocrine stress responses and improve intraoperative hemodynamic stability without any delay in recovery from general anesthesia [1-3]. Remifentanil-treated patients have been reported to exhibit lower intraoperative systolic and diastolic blood pressure (DBP) than fentanyl-treated patients [3], suggesting that remifentanil may decrease intraoperative blood loss. We therefore compared estimated intraoperative blood loss during spinal surgery between patients administered remifentanil or fentanyl as an opioid adjuvant to general anesthesia. In addition, indices of intraoperative hemodynamic stability were compared, including heart rate and BP changes during anesthesia onset, skin incision, laminoplasty or laminectomy, and anesthesia recovery.

## Methods

The study was approved by the Ethics Committee of National Hospital Organization Zentsuji Hospital, and the need for informed consent was waived. We retrospectively reviewed the records all patients who underwent spinal surgery (laminoplasty or laminectomy) under general anesthesia at National Hospital Organization Zentsuji Hospital between April 2010 and March 2011. Patients who underwent spinal fusion surgery, patients on hemodialysis, and patients who received induced hypotensive anesthesia were excluded. All operations were performed by the same surgeon. No preanesthetic medication was administered to these patients. All patients studied received remifentanil or fentanyl in combination with sevoflurane (with or without nitrous oxide) for general anesthesia, and no other opioids were administered except remifentanil and fentanyl. Demographic data, including age, gender, height, weight, ASA physical status, and history of hypertension, were recorded for each patient. Surgical data recorded included duration of anesthesia and operation time, type of surgery, number of decompression segments, total doses of remifentanil and fentanyl, total doses of ephedrine and nicardipine, intravascular fluid volume, urine output, temperature, and the following hemodynamic indices: heart rate (HR), systolic BP (SBP), mean BP (MBP), and DBP. These hemodynamic parameters were recorded at the following time points: Tb, before induction of anesthesia; T0, at skin incision; T30, 30 min after skin incision; T60, 60 min after skin incision; T90, 90 min after skin incision; and Te, the end of anesthesia. Laboratory levels of preoperative and postoperative hemoglobin, hematocrit, and platelet count were also obtained.

We divided the patients into two groups, a remifentanil group and a fentanyl group. In the remifentanil group, remifentanil was administered by continuous infusion for intraoperative analgesia, and fentanyl was administered for transitional analgesia. In the fentanyl group, fentanyl was administered at bolus doses for intraoperative analgesia. The infusion rate of remifentanil or the dose of fentanyl during maintenance was left to the discretion of the attending anesthesiologist. The primary end point was the estimated intraoperative blood loss, which was calculated by factoring in the surgical suction volume and the weight of the gauze from the operative field. Blood loss estimates from the floor and surgical gowns and drapes were not included.

Statistical analyses were performed using SPSS version 18 software (SPSS, Inc., Chicago, IL). Continuous variables were compared by unpaired Student’s t-tests. Categorical variables were analyzed with χ^2^ or Fisher’s exact tests where appropriate. For hemodynamic variables, two-way repeated-measures analysis of variance (ANOVA) followed by Bonferroni *post hoc* tests were performed to evaluate the effects of time of analgesia, anesthetic group, and time × group interactions. Data are expressed as number of patients or mean ± standard deviation. Statistical significance was set at *P* < 0.05.

## Results

Sixty-eight patients who underwent spinal surgery (laminoplasty or laminectomy) during the review period were included, whereas four were excluded. These included three hemodialysis patients and one patient who received induced hypotensive anesthesia. Of the 64 patients accepted, 35 had received remifentanil (remifentanil group) and 29 had received fentanyl (fentanyl group) as an opioid adjuvant during general anesthesia.

There were no significant differences in the demographic variables including age, gender ratio, weight, height, body mass index, ASA physical status, and history of hypertension between anesthetic groups (Table 1). Similarly, there were not significant differences in the intraoperative variables duration of anesthesia, operation time, site of surgery (cervical vs. lumbar spine), number of decompression segments, intravascular fluid volume, and body temperature between the two groups (Table 2). Total dose of intraoperative fentanyl was significantly greater in the fentanyl group than in the remifentanil group (272 ± 79 μg vs. 112 ± 74 μg, *P* < 0.001) (Table 2).

**Table 1:** Patient demographics

	**Remifentanil (n = 35)**	**Fentanyl (n = 29)**
Age (years)	75 ± 9	74 ± 8
Sex (M/F)	21/14	13/16
Height (cm)	155 ± 10	152 ± 9
Weight (kg)	56 ± 10	56 ± 11
ASA physical status (I/II/III)	1/24/10	2/21/6
History of hypertension (n)	22	20

Data presented as mean ± SD or number of patients.

There was no statistically significant difference between the groups.

**Table 2:** Surgery/anesthesia-related parameters

	**Remifentanil (n = 35)**	**Fentanyl (n = 29)**
Duration of anesthesia (min)	212 ± 44	220 ± 43
Duration of surgery (min)	158 ± 44	159 ± 42
Anesthetics		
Remifentanil (mg)	3.2 ± 1.1	
Fentanyl (μg)	112 ± 74*	272 ± 79
Site of surgery (n)		
Cervical spine	19	17
Lumbar spine	16	12
Number of decompression segments (n)	3.1 ± 1.5	3.6 ± 1.5
Amount of ephedrine (mg)	8.3 ± 7.3*	3.3 ± 4.6
Amount of nicardipine (mg)	0 ± 0*	0.3 ± 0.7
Temperature (°C)	37.0 ± 0.7	36.8 ± 0.6
Fluid volume (mL)	1146 ± 314	1050 ± 244
Urine output (mL)	324 ± 377	293 ± 192
Blood loss (mL)	125 ± 67*	165 ± 82

Data presented as mean ± SD or number of patients.

*Statistically significant difference from the fentanyl group (*P* < 0.05).

Intraoperative blood loss was significantly lower in the remifentanil group than in the fentanyl group (125 ± 67 mL vs. 165 ± 82 mL, *P* = 0.035) (Table 2). The total amount of ephedrine administered was higher in the remifentanil group than in the fentanyl group (8.3 ± 7.3 mg vs. 3.3 ± 4.6 mg, *P* = 0.002) (Table 2). More nicardipine was used in the fentanyl group than in the remifentanil group (0.3 ± 0.7 mg vs. 0 ± 0 mg, *P* = 0.005) (Table 2).

Preoperative laboratory variables were comparable between the two groups (Table 3). Postoperative hemoglobin and hematocrit levels were lower in the remifentanil group than in the fentanyl group, but platelet count was not significantly different (Table 3).

**Table 3:** Perioperative data

	**Remifentanil (n = 35)**	**Fentanyl (n = 29)**
Hemoglobin (g/dL)		
Preoperative	12.4 ± 2.0	12.9 ± 1.8
Postoperative	11.1 ± 1.6*	12.0 ± 1.8
Hematocrit (%)		
Preoperative	37.1 ± 5.4	38.5 ± 5.1
Postoperative	33.0 ± 4.5*	35.9 ± 5.0
Platelets (× 10^4^/mm^3^)		
Preoperative	23.6 ± 6.1	21.8 ± 3.8
Postoperative	20.4 ± 5.1	18.3 ± 3.9

Data presented as mean ± SD.

^*^Statistically significant difference from the fentanyl group (*P* < 0.05).

**Figure 1:**
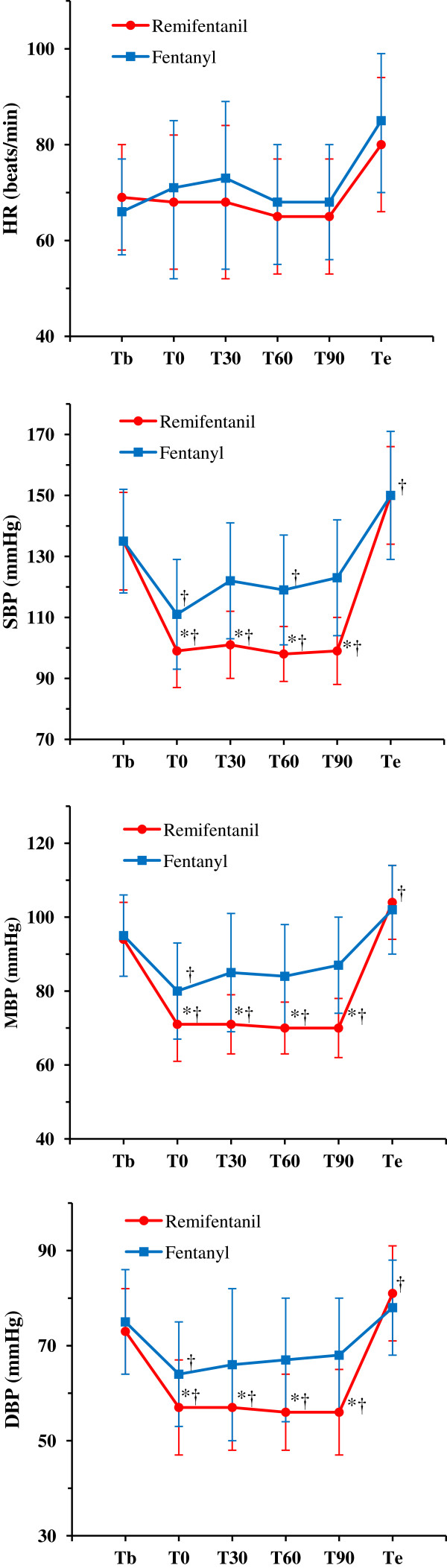
**Hemodynamic measurements.** Data presented as mean ± SD. * Statistically significant difference from the fentanyl group (*P* < 0.05), † Statistically significant difference from baseline in the same group (*P* < 0.05). *Tb* before induction of anesthesia, *T0* skin incision, *T30* 30 min after skin incision, *T60* 60 min after skin incision, *T90* 90 min after skin incision, *Te* the end of anesthesia, *HR* heart rate, *SBP* systolic blood pressure, *MBP* mean blood pressure, *DBP* diastolic blood pressure.

The hemodynamic variables HR, SBP, MBP, and DBP were compared both within anesthesia groups before, during, and after surgery as well as between analgesia groups (Figure 1). There was no significant change in heart rate over the entire perioperative period in either anesthesia group, and no significant difference in HR between groups at any perioperative time point. In both anesthesia groups, SBP, MBP, and DBP decreased significantly at skin incision, but returned to and then exceeded baseline by the end of anesthesia. Intraoperative SBP, MBP, and DBP were lower in the remifentanil group at all intraoperative measurement times (*P* < 0.05 for all hemodynamic parameters), suggesting that remifentanil may decrease intraoperative blood loss by inducing a sustained drop in BP during the intraoperative period.

## Discussion

In this study, we demonstrated that administration of remifentanil during general anesthesia significantly decreased intraoperative blood loss compared with that of fentanyl. This is the first study to show that the selection of adjuvant opioid analgesic significantly influences intraoperative blood loss during spinal surgery.

Remifentanil has several advantages over other opioids (i.e., fentanyl, alfentanil, or sufentanil) used during general anesthesia, including promotion of hemodynamic stability and very rapid onset and recovery. For example, Philip et al. [1] reported that remifentanil provided better intraoperative stability than alfentanil in patients undergoing ambulatory laparoscopic procedures, as indicated by fewer hemodynamic response to intubation and trocar insertion. Twersky et al. [3] reported a more stable intraoperative course and faster emergence after remifentanil administration than fentanyl administration in a large population of surgical patients. Moreover, remifentanil-treated patients exhibited lower intraoperative systolic and DBP (by 10–15 mmHg) as well as lower intraoperative heart rate (by 10–15 bpm) than fentanyl-treated patients without an increase in significant adverse events.

Although intraoperative hemodynamic stability can be achieved by administration of relatively large doses of any anesthetic agent, such treatment may delay extubation or recovery, particularly the time until patients can response to queries posed by the clinicians. Furthermore, delayed awakening from anesthesia may complicate postoperative neurological assessment after spinal surgery. Times to patient response, extubation, and initiation of spontaneous ventilation were all significantly shorter in remifentanil-treated patients than in surgery patients treated with other opioids [4], likely because remifentanil is eliminated more rapidly from the blood. Thus, remifentanil stabilizes intraoperative hemodynamics without delaying recovery. However, these previous studies focused on hemodynamic changes associated with surgical stress rather than on the effects of different opioids on intraoperative bleeding.

Consistent with several previous studies, remifentanil-treated patients exhibited 10–20 mmHg lower intraoperative SBP, MBP, and DBP than fentanyl-treated patients at all intraoperative measurement points. In addition, more ephedrine was used in the remifentanil group than in the fentanyl group, and more nicardipine was used in the fentanyl group than in the remifentanil group, indicating that continuous infusion of remifentanil cause a greater suppression of the endocrine stress and inflammatory responses than intermittent boluses of fentanyl. Winterhalter et al. [5] reported that perioperative endocrine stress responses, including increases in plasma epinephrine and norepinephrine levels, were attenuated in patients receiving continuous remifentanil infusion compared with those in patients receiving intermittent fentanyl during general anesthesia for coronary artery bypass grafting. Thus, remifentanil may improve intraoperative hemodynamic stability by attenuating the endocrine stress reaction.

Intraoperative blood loss is a major concern for both surgeons and anesthesiologists. Decreased bleeding enhances the clarity of the surgical field, which can decrease intraoperative and anesthesia times. Indeed, it was reported that a bloodless surgical field decreased the time required for vertebral disc resection [6]. Greater blood loss also increases the requirement for blood transfusions, and several reports have suggested that allogeneic blood transfusions are a risk factor for postoperative bacterial infections [7,8]. It has been demonstrated that the amount of bleeding during surgery is strongly dependent on arterial BP [9]. Induced hypotension has long been used as an effective method for decreasing intraoperative blood loss during spinal surgery. Agents used to induce and maintain intraoperative hypotension include volatile anesthetics (sevoflurane, isoflurane, and desflurane), intravenous anesthetics (propofol and thiopental), sodium nitroprusside, nitroglycerin, calcium channel antagonists, and beta-blocking agents. Epidural anesthesia has also been shown to decrease intraoperative blood loss [10]. In contrast to induced hypotension using volatile anesthetics, the effect of the intraoperative administration of opioid analgesics on blood loss was not previously examined. We suggest that administration of remifentanil during general anesthesia decreases intraoperative blood loss, at least compared with fentanyl administration, during spinal surgery.

In contrast to studies associating intraoperative blood loss with arterial BP, two previous reports concluded that susceptibility to surgical bleeding during posterior spinal surgery under normotensive anesthesia was affected by vertebral intraosseous pressure but not by systemic arterial BP [10,11]. According to Kakiuchi [11], systemic arterial BP did not correlate with vertebral intraosseous pressure, implying that patients with low arterial BP do not necessarily have a low intraosseous pressure. In the present study, only arterial BP was measured; therefore, further studies are required to confirm whether remifentanil attenuates intraoperative bleeding by decreasing arterial BP, intraosseous pressure, or both.

This study shares the major limitations of retrospective studies. Specifically, data were obtained from medical records that were not specifically designed to address the relationship between intraoperative opioid anesthetic administration and blood loss. For the analysis of intraoperative blood loss, laboratory levels of postoperative hemoglobin, hematocrit, and platelet count should ideally be measured just after the surgery. In our patients, however, the time point of blood sampling was irregular. These values may thus reflect both intraoperative and postoperative blood loss. In addition, the bispectral index was not available as indicator of the level of consciousness during general anesthesia; therefore, decreased blood loss may have depended, at least in part, on differences in the dose of sevoflurane. However, it has been shown that sevoflurane dosage was significantly lower in patients who received remifentanil as an opioid adjuvant to general anesthesia instead of fentanyl [12]. Therefore, we propose that the enhanced intraoperative hemodynamic stability observed in the present study was because of administration of remifentanil.

## Conclusions

This study demonstrates that intraoperative blood loss during spinal surgery can be decreased by using remifentanil rather than fentanyl as the opioid adjuvant during general anesthesia. Given the importance of decreasing intraoperative bleeding on clinical outcome, the effect of remifentanil on blood loss warrant a large-scale prospective randomized controlled trial. In addition, further studies are required to investigate whether our findings are applicable to other surgical procedures.

## Competing interests

The authors declare that they have no competing interests.

## Authors’ contributions

HK designed the study and collected the data, analyzed the data, and wrote the manuscript. SM, TM and EH collected the data. MK analyzed the data. FT and SO helped to design the study. All authors read and approved the final manuscript.

## Pre-publication history

The pre-publication history for this paper can be accessed here:

http://www.biomedcentral.com/1471-2253/13/46/prepub
